# Increased intrapharyngeal pressure with combined use of high-flow nasal cannula and a surgical face mask: a preliminary study

**DOI:** 10.20407/fmj.2019-004

**Published:** 2019-09-25

**Authors:** Satoshi Komatsu, Yoshitaka Hara, Naohide Kuriyama, Tomoyuki Nakamura, Chizuru Yamashita, Hidefumi Komura, Junpei Shibata, Osamu Nishida

**Affiliations:** Department of Anesthesiology and Critical Care Medicine, Fujita Health University, School of Medicine, Toyoake, Aichi, Japan

**Keywords:** Continuous positive airway pressure, High-flow therapy, Intensive care unit, Respiratory failure, Volunteer

## Abstract

**Objectives:**

Nasal high-flow (NHF) therapy provides continuous positive airway pressure (CPAP), flushes the anatomical dead space, and improves mucociliary clearance. CPAP is usually applied at a flow rate at or above an established threshold value with the mouth closed because it is hard to maintain it with an open mouth. We conducted a prospective study to validate our hypothesis that CPAP can be applied with the mouth open through a surgical face mask.

**Methods:**

We inserted 12-Fr nasogastric tubes through the noses of 18 healthy individuals and fixed each tube within the pharynx to monitor the intrapharyngeal pressure. We monitored the pressure during the following two conditions: NHF oxygen with the mouth open (condition O) and NHF oxygen with the mouth open and wearing a surgical face mask (condition OM). We set the NHF rate at 40 L/min and the oxygen concentration at 21%, under all conditions. We measured the intrapharyngeal pressure five times during each inspiration and expiration, and calculated mean values.

**Results:**

The mean expiratory intrapharyngeal pressure (median [interquartile range]) increased significantly from the baseline during conditions O (2.08 [1.58–4.02] cm H_2_O) and OM (3.35 [2.72–3.79] cm H_2_O). In addition, there was a significant difference in pressure between conditions O and OM (p=0.0263, Wilcoxon signed-rank test).

**Conclusions:**

In our healthy volunteers, the intrapharyngeal pressures increased during expiration with an open mouth while wearing a surgical face mask.

## Introduction

High-flow therapy (HFT) is widely used in neonatal and pediatric care,^1^ cardiology,^2^ respiratory medicine,^3^ and intensive care^4^ HFT improves the quality of life, enables oral intake, provides a highly accurate fraction of inspired oxygen (F_I_O_2_) with a high-flow rate and continuous positive airway pressure (CPAP), improves mucociliary clearance, and flushes the anatomical dead space. In this study, we focused on airway pressure because the intrapharyngeal pressure is significantly higher when the mouth is closed.^5^ In another study, intrapharyngeal pressure changes were measured in healthy individuals with open and closed mouths and it was found that the airway pressure with a 40 L/min nasal high-flow (NHF) rate was 2.2 cm H_2_O with the mouth open and 5.5 cm H_2_O with the mouth closed.^6^ However, few patients are able to keep their mouths closed in clinical settings, particularly in the intensive care unit (ICU). Some studies have investigated changes in intrapharyngeal pressure during NHF use,^5,6^ but none have tried to improve its therapeutic effects. We hypothesized that patients receiving NHF could approximate closed-mouth intrapharyngeal pressures if wearing a surgical face mask and designed an interventional, prospective study to assess the effect of surgical face masks on the airway pressure during NHF.

## Methods

### Study design

This was an interventional, prospective study (UMIN CTG 000023307).

### Subjects

Eighteen healthy individuals (nine men, nine women) participated in this study.

### Measurement methods

We inserted a 12-Fr enteral feeding tube through each participant’s nose (JF-C13230Q, JMS, Hiroshima, Japan) with the tip resting in the pharynx to monitor their intrapharyngeal pressures. We connected the tube to a pressure line and transducer set (LSK366PSC, 550D), Argon, Singapore] and measured the intrapharyngeal pressure using a biomonitor (CU-191R, Nihon Kohden, Tokyo, Japan). We obtained measurements using a pressure line filled with air, and identified pressure changes secondary to inspiration and expiration by designating the minimum value as the inspiration pressure and the maximum value as the expiration pressure. The surgical face masks used were manufactured by Nissho Sangyo (Tokyo, Japan).

### Data collection and selection methods

**Figure 1:**
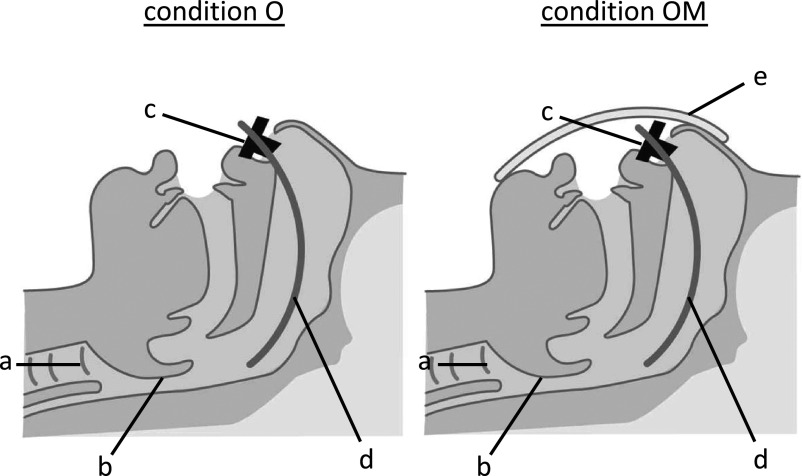
Sagittal representation of two situations: “open mouth during NHF” (condition O) and “open mouth while wearing a surgical face mask during NHF” (condition OM). a: trachea, b: epiglottis, c: nasal cannula, d: nasopharyngeal tube, e: surgical face mask

We used an Optiflow NHF cannula and a MR850 heated humidifier (Fisher & Paykel Healthcare, Auckland, New Zealand) and set the NHF at F_I_O_2_ 0.21 and the flow rate at 40 L/min. We asked the participants to breathe five times in succession under each of the following two situations: open mouth during NHF” (condition O) and “open mouth while wearing surgical face mask during NHF” (condition OM) (Figure 1). We gave no specific instructions, thus allowing the participants to breathe freely. We used large nasal cannulas for men and medium sized ones for women. The procedure lasted approximately 30 min per person.

We calculated mean values by excluding the minimum and maximum values and calculating the mean of the three remaining values.

### Ethical considerations

We thoroughly explained the study to the healthy volunteers, who provided written informed consent before participating. The Institutional Review Board of Fujita Health University approved this study and we registered it as a UMIN clinical trial (Ethics Review Committee, Fujita Health University 15-173, UMIN CTG 000023307: https://upload.umin.ac.jp/cgi-open-bin/ctr_e/ctr_view.cgi?recptno=R000024258).

### Statistical analysis

We have here expressed age, height, and weight as means and standard deviations and values for other variables as medians and interquartile ranges. We made two-group comparisons using the Wilcoxon signed-rank test. We performed all statistical analyses with EZR (Saitama Medical Center, Jichi Medical University, Saitama, Japan), which is a graphical user interface for R (The R Foundation for Statistical Computing, Vienna, Austria). More precisely, it is a modified version of R commander designed to add statistical functions frequently used in biostatistics.

## Results

**Figure 2:**
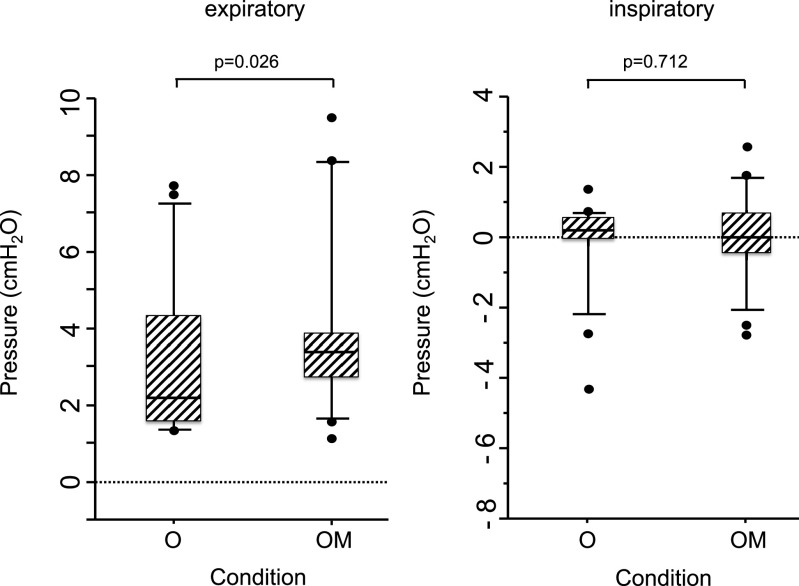
Mean expiratory and inspiratory intrapharyngeal pressure in healthy individuals (n=18). The two conditions were “open mouth during NHF” (condition O), and “open mouth while wearing a surgical face mask during NHF” (condition OM). Mean expiratory intrapharyngeal pressure in healthy volunteers (n=18) was calculated for O (2.09 [1.59–4.02] cm H_2_O) and OM (3.35 [2.72–3.80] cm H_2_O) conditions. Pressure was differed significantly between these conditions (p=0.0263). Mean inspiratory intrapharyngeal pressures were calculated for O (0.18 [0–0.55] cm H_2_O) and OM (0 [–0.34–0.62] cm H_2_O) conditions.

We enrolled 18 healthy volunteers with a mean age of 27±4.07 years, mean height 164±8.03 cm, mean weight 57.6±7.65 kg, and mean body mass index (BMI) 21.2±1.90. We calculated mean expiratory intrapharyngeal pressures during conditions O (2.09 [1.59–4.02] cm H_2_O) and OM (3.35 [2.72–3.80] cm H_2_O). We found that the pressures were differed significantly between conditions O and OM (p=0.0263). We also calculated the mean inspiratory intrapharyngeal pressures for conditions O (0.18 [0–0.55] cm H_2_O) and OM (0 [–0.34–0.62] cm H_2_O) (Figure 2).

## Discussion

In our volunteers, the intrapharyngeal pressure increased during expiration with an open mouth while wearing a surgical face mask. Although this increase in intrapharyngeal pressure did not reach the levels measured when the participants were breathing with their mouths closed, it was close to that. Because our study participants were healthy volunteers, we cannot assume the same findings in the clinical setting. Many patients in ICU have narrow nasal cavities because they have had tubes for feeding or drainage inserted into them. There is also a tendency for many patients in ICU to breathe through the mouth even when awake. A previous report^7^ have shown that the pharyngeal pressure does not differ between nasal breathing and oral breathing in individuals who are awake, and it has been suggested that using a surgical face mask in such patients may have certain effects. Furthermore, although it is known that upper airway resistance increases in sleeping patients, during NHF the CPAP effect may reduce the patient’s inspiratory effort.^7^ However, this requires further study. Our findings concerning intrapharyngeal pressure changes during inspiration suggest that negative pressure builds in the pharynx when the mouth is closed, even during NHF administration at 40 L/min. With the mouth open, the intrapharyngeal pressure rarely reached negative levels, regardless of whether or not a surgical face mask was being worn. This suggests that inspiration is close to effortless when the mouth is open. In addition, even with an open mouth, use of a surgical face mask enabled maintenance of the CPAP during expiration, suggesting this effect occurs during both inspiration and expiration when wearing a surgical face mask while receiving NHF (even without keeping the mouth closed). NHF is considered to have less CPAP effect than noninvasive positive pressure ventilation (NPPV).^8^ It may be preferable to uses NPPV therapy to ensure positive airway pressure during expiration. However, it may be possible to enhance the effect of treatment simply and relatively easily by achieving an increase in pharyngeal pressure at the end of expiration.

When HFT was first introduced in our facility, we initiated it at a rate of 40 L/min because our patients were experiencing extreme discomfort at rates of 50 L/min or higher. Therefore, we did not test different flow rates. However, in their study of healthy individuals, Groves et al. reported that intrapharyngeal pressures increase as flow rates increase and that the increases are larger with a closed than with an open mouth.^6^ Thus, we anticipated that flow rate increases would have similar effects in individuals wearing a surgical face mask.

Ideally CPAP effects should be determined by measuring alveolar pressure changes, however, measuring these is difficult; we therefore measured intrapharyngeal pressures in lieu of intra-alveolar pressures on the basis of reasoning similar to that of other investigators.^6,9,10^

We are aware that our study has some limitations. Our sample size is small (n=18), which limits the generalizability of our results. The participants were all healthy young adults and thus did not necessarily reflect individuals in a clinical setting, in whom the combined use of a high-flow nasal cannula and surgical face mask may produce different results.

Surgical face masks are inexpensive and reliably effective. We recommend their proactive use during safe and simple procedures. Other researchers have studied the effects of and indications for NHF, whereas we examined means of increasing the clinical effectiveness of this therapy.

In our volunteers, intrapharyngeal pressures increased during expiration with an open mouth while wearing a surgical face mask. Our results suggest that wearing a surgical face mask during NHF therapy may amplify the therapeutic effects of CPAP.

## References

[1] Yoder BA, Stoddard RA, Li M, King J, Dirnberger DR, Abbasi S. Heated, humidified high-flow nasal cannula versus nasal CPAP for respiratory support in neonates. Pediatrics 2013; 131: e1482–1490. 23610207 10.1542/peds.2012-2742

[2] Roca O, Pérez-Terán P, Masclans JR, Pérez L, Galve E, Evangelista A, Rello J. Patients with New York Heart Association class III heart failure may benefit with high flow nasal cannula supportive therapy: high flow nasal cannula in heart failure. J Crit Care 2013; 28: 741–746. 23602035 10.1016/j.jcrc.2013.02.007

[3] Sztrymf B, Messika J, Mayot T, Lenglet H, Dreyfuss D, Ricard JD. Impact of high-flow nasal cannula oxygen therapy on intensive care unit patients with acute respiratory failure: a prospective observational study. J Crit Care 2012; 27: 324.e9–13. 10.1016/j.jcrc.2011.07.075 21958974

[4] Sztrymf B, Messika J, Bertrand F, Hurel D, Leon R, Dreyfuss D, Ricard JD. Beneficial effects of humidified high flow nasal oxygen in critical care patients: a prospective pilot study. Intensive Care Med 2011; 37: 1780–1786. 21946925 10.1007/s00134-011-2354-6

[5] Parke R, McGuinness S, Eccleston M. Nasal high-flow therapy delivers low level positive airway pressure. Br J Anaesth 2009; 103: 886–890. 19846404 10.1093/bja/aep280 PMC2777940

[6] Groves N, Tobin A. High flow nasal oxygen generates positive airway pressure in adult volunteers. Aust Crit Care 2007; 20: 126–131. 17931878 10.1016/j.aucc.2007.08.001

[7] Fitzpatrick MF, McLean H, Urton AM, Tan A, O’Donnell D, Driver HS. Effect of nasal or oral breathing route on upper airway resistance during sleep. Eur Respir J 2003; 22: 827–832. 14621092 10.1183/09031936.03.00047903

[8] Parke RL, McGuinness SP. Pressures delivered by nasal high flow oxygen during all phases of the respiratory cycle. Respir Care 2013; 58: 1621–1624. 23513246 10.4187/respcare.02358

[9] Corley A, Caruana LR, Barnett AG, Tronstad O, Fraser JF. Oxygen delivery through high-flow nasal cannulae increase end-expiratory lung volume and reduce respiratory rate in post-cardiac surgical patients. Br J Anaesth 2011; 107: 998–1004. 21908497 10.1093/bja/aer265

[10] Parke RL, Eccleston ML, McGuinness SP. The effects of flow on airway pressure during nasal high-flow oxygen therapy. Respir Care 2011; 56: 1151–1155. 21496369 10.4187/respcare.01106

